# ONS Health Insight Survey in general practice: insight, not oversight

**DOI:** 10.3399/BJGP.2026.0195

**Published:** 2026-06-01

**Authors:** James Routledge, Sarah Opie-Martin, Luisa M Pettigrew

**Affiliations:** 1 The Health Foundation, London, UK; 2 Clinical Fellow, London School of Hygiene & Tropical Medicine, London, UK

Patient experience is a defining measure of quality in the NHS, and the 10 Year Health Plan for England has committed to embedding patient-reported experience and outcomes into national indicators by 2029.^1^ The GP Patient Survey (GPPS) remains the long-established annual measure of experiences of primary care services in England.^2^ However, in addition, the Office for National Statistics (ONS) launched the Health Insight Survey in July 2024 to provide data on experiences of general practice, dental care, pharmacies, NHS community services, and hospital waiting lists.

The HIS is now increasingly being referenced by policymakers. Results from the HIS are being used in government communications to illustrate improvements in satisfaction with general practice.^3,4^ NHS England’s *Priorities and Operational Planning Guidance* and *Medium Term Planning Framework* propose using HIS data to assess integrated care boards’ (ICBs) performance on access to general practice.^5,6^ It is therefore important to understand the strengths and limitations of the HIS.

## What is the Health Insight Survey?

The HIS is a 4-weekly online survey of people living in England aged ≥16 years, run by the ONS and commissioned by NHS England — participants also have the option to complete it by telephone. The sample for the survey was originally drawn from the approximately 240 000 participants in the ONS COVID-19 Infection Survey and limited to people living in private households. It is published as *‘official statistics in development’*, signalling that methods and outputs may evolve.^7^ Typically, between 75 000 and 90 000 people complete the survey, and the results are published the month following collection.^8^ Full quality and methods information for the HIS can be found on the ONS website,^7^ and frequently asked questions about the GPPS including how it relates to the HIS are available online.^9^

The survey focuses on people who have recently used or attempted to use NHS services, asking about experiences within the last 28 days. This narrow recall period allows the survey to identify short-term changes in behaviour and access, seasonal variation, and early signals of policy effects.

## Caution is needed when interpreting results

While the sample size of the HIS is relatively large, it has methodological limitations that may affect its results. People who join and remain engaged in online panel-based studies (surveys of the same individuals over time) tend to be more digitally confident and more responsive to surveys,^10^ and the profile of HIS responders differs from that of the general population.

In Wave 20 of the survey, published in February 2026, 56% of responders were women (compared with 52% nationally), 26% came from a rural town or village (compared with 17% nationally — noting this figure includes people aged <16 years ), 60% were aged ≥65 years (compared with 23% nationally), 38% had a long-term condition (compared to 46% in the 2024 Health Survey for England, although this figure varies by source), 95% were White (compared with 83% nationally), and 7% were from the most deprived quintile whereas 31% were from the least deprived quintile.^7,11^ Previous analysis of the GPPS shows that older, White, and less deprived groups typically report more positive experiences of the NHS, meaning that the HIS is likely to produce more favourable absolute estimates than surveys that capture a broader demographic mix.^12^ Gender differences and the presence of long-term conditions influence the use of general practice itself, and rural responders are less likely than those from urban areas to report negative experiences of general practice.^13,14^

To address underrepresentation, weighting is used by the HIS to make the achieved sample more closely resemble the wider population.^7^ The survey also periodically undertakes targeted ‘boosts’ to increase the number of responses from underrepresented groups, including from care homes and other communal establishments. Following this, re-weighting is needed to ensure any changes in trends are not due to variability in the sample population. While these steps improve the accuracy of the estimates, statistical uncertainty remains high, making it difficult to identify meaningful differences between groups. In addition, the 28-day recall makes it more likely that responders report on satisfaction related to interactions that happen more frequently, such as arranging online prescriptions, rather than for making an appointment — which may mask differences between these in terms of satisfaction.

In February 2026, across 42 ICBs there was an average of <2000 responders per ICB, however numbers ranged between ~800 and >4000. While these differences may reflect different ICB sizes, unevenly distributed samples produce wide confidence intervals, meaning that apparent differences between ICBs may reflect sampling variation rather than real differences in performance. Attrition, where participants drop out, is a common issue with panel-based surveys. The response rate of the HIS has fallen over time, from ~43% initially to ~29% more recently, and in parallel non-responders have been removed from the sample. While the total number of participants has remained relatively stable by using boosts, changes to the population sampled has reduced its utility to track changes in attitudes within the original cohort. Repeated participation can also change how people behave or answer questions, known as ‘panel conditioning’, because prior survey experience may introduce bias by altering true or reported responses.^10^

Taken together, these design constraints warrant caution: they increase the likelihood of more positive overall ratings given the demographic profile of responders, limit generalisability, and reduce the visibility of meaningful local variation in patient experience.

## Insight, not oversight

These methodological characteristics matter because if HIS findings are interpreted without sufficient attention to how the survey is constructed, they may be taken as evidence of improvement or decline when the true picture is more complex.

Using HIS results to performance-manage ICBs could have unintended consequences for general practice. For example, if an ICB appears to be underperforming due to the composition of the HIS sample, the ICB may ask all local practices respond to performance metrics that may not reflect their patients’ needs or local context. Conversely, practices serving more deprived or diverse populations may face substantial access challenges that are invisible in HIS data if their patients are not participating. Treating results as comparable across areas risks obscuring important variation and could lead to misdirected interventions. It is therefore important that the HIS in its current format is used, as the name suggests, to provide insight, not oversight, and that it is used alongside other measures of patient satisfaction and quality of care.

## Using both surveys to support better care

In contrast to the HIS, the GPPS is an annual survey sent to a random sample of patients aged ≥16 years who are registered with a GP practice in England and have been continuously registered for at least 6 months. In 2025, 2.72 million patients were invited to take part and >700 000 responses were received; a 26% response rate.^2^ The survey aims to secure at least 100 responses per practice and 200 per primary care network (PCN), enabling reporting at practice and PCN levels. It uses a mixed-mode approach, offering postal and online completion, telephone options, and accessible formats, with multiple reminders issued across 3 months of fieldwork. These features help ensure the inclusion of people who are digitally excluded or have limited time, including those working full-time or with caring responsibilities. Results are usually published 3 months following the completion of data collection. The methods used by the GPPS are consistent with best practice in surveys that aim to generate a representative understanding of experiences. It does, however, come at a cost, with the last 5-year contract being priced at £19.9 million.^15^

The HIS can complement the GPPS by providing more frequent insights and providing greater logistical flexibility. It can highlight early shifts in behaviour within its defined responder group and flag issues that may warrant deeper investigation. However, using it to performance-manage ICBs or by ministers as evidence of policy success risks overlooking the experiences of parts of the population and overstating what the survey can reliably show.
